# A “terminal” case of glycan catabolism: Structural and enzymatic characterization of the sialidases of *Clostridium perfringens*

**DOI:** 10.1016/j.jbc.2024.107750

**Published:** 2024-09-07

**Authors:** Brendon J. Medley, Kristin E. Low, Jackline D.W. Irungu, Linus Kipchumba, Parandis Daneshgar, Lin Liu, Jolene M. Garber, Leeann Klassen, G. Douglas Inglis, Geert-Jan Boons, Wesley F. Zandberg, D. Wade Abbott, Alisdair B. Boraston

**Affiliations:** 1 Department of Biochemistry & Microbiology, University of Victoria, Victoria, British Columbia, Canada; 2 Agriculture and Agri-Food Canada, Lethbridge Research and Development Centre, Lethbridge, Alberta, Canada; 3 Department of Chemistry, Irving K. Barber Faculty of Science, University of British Columbia, Kelowna, British Columbia, Canada; 4 Complex Carbohydrate Research Center, University of Georgia, Athens, Georgia, USA; 5 Chemical Biology and Drug Discovery, Utrecht University, Utrecht, The Netherlands

**Keywords:** sialidase, glycoside hydrolase, neuraminic acid, Clostridium perfringens, carbohydrate-processing, inhibitor, X-ray crystal structure

## Abstract

Sialic acids are commonly found on the terminal ends of biologically important carbohydrates, including intestinal mucin *O*-linked glycans. Pathogens such as *Clostridium perfringens*, the causative agent of necrotic enteritis in poultry and humans, have the ability to degrade host mucins and colonize the mucus layer, which involves removal of the terminal sialic acid by carbohydrate-active enzymes (CAZymes). Here, we present the structural and biochemical characterization of the GH33 catalytic domains of the three sialidases of *C. perfringens* and probe their substrate specificity. The catalytically active domains, which we refer to as NanH_GH33_, NanJ_GH33_, and NanI_GH33_, displayed differential activity on various naturally occurring forms of sialic acid. We report the X-ray crystal structures of these domains in complex with relevant sialic acid variants revealing the molecular basis of how each catalytic domain accommodates different sialic acids. NanH_GH33_ displays a distinct preference for α-2,3-linked sialic acid, but can process α-2,6-linked sialic acid. NanJ_GH33_ and NanI_GH33_ both exhibit the ability to process α-2,3- and α-2,6-linked sialic acid without any significant apparent preference. All three enzymes were sensitive to generic and commercially available sialidase inhibitors, which impeded sialidase activity in cultures as well as the growth of *C. perfringens* on sialylated glycans. The knowledge gained in these studies can be applied to *in vivo* models for *C. perfringens* growth and metabolism of mucin *O*-glycans, with a view toward future mitigation of bacterial colonization and infection of intestinal tissues.

Sialic acids are commonly found on the terminal ends of biologically important carbohydrates (1). They are abundant in the mucin glycans of humans and animals, mammalian milk oligosaccharides, bacterial surfaces, and the extracellular matrices of marine biofilms (2, 3, 4). Within the gastrointestinal (GI) tract, the microbial catabolism of sialic acid, which is typically harvested from host mucin glycans, can confer advantages to select groups of commensal bacteria commonly overrepresented during microbiome dysbiosis, such as *Escherichia coli* (5) and *Ruminococcus gnavus* (6). This process can influence the virulence of bacterial pathogens and contribute to inflammation during disease (7, 8, 9, 10, 11). *Clostridium perfringens*, an opportunistic bacterial pathogen that commonly inhabits animal and human GI tracts, is one of the earliest known microbes to produce sialidase(s), also referred to as neuraminidases, that are capable of processing sialylated glycoconjugates [for example see (12)]. Abundant genomic evidence now indicates that the genomes of all *C. perfringens* strains contain at least one sialidase encoding gene with many strains containing up to three. The sialidases have shown properties consistent with contributing to adherence to and colonization of the mammalian GI mucosa (13, 14, 15, 16). The ability of poultry-adapted strains of *C. perfringens* to degrade host mucin through sialic acid metabolism is also predicted to have a major role in virulence, specifically in poultry necrotic enteritis (NE) (17). NE results in billion-dollar losses annually to the poultry industry (18), particularly following the phasing out of prophylactic antimicrobial use in the livestock industry. Thus, further understanding the metabolism of sialic acid by *C. perfringens* may aid efforts to develop alternative strategies for managing the disease (19).

Recent work has demonstrated that when *C. perfringens* CP4, a poultry NE-causing strain, is grown in the presence of mucins a statistically significant decrease in sialylated and sulfated mucin *O*-glycans occurs and corresponds with increases in several neutral glycan species (17). Upon quantification of the two forms of sialic acids *N*-acetylneuraminic acid (Neu5Ac) and *N*-glycolylneuraminic acid (Neu5Gc), a specific and significant decrease in Neu5Ac was seen, suggesting it was being preferentially consumed by the pathogen. The large repertoire of carbohydrate-active enzymes (CAZymes) within the genome of *C. perfringens* strains positions this bacterium to dismantle host mucin *O*-glycans effectively and efficiently. It has been postulated that the removal of terminal sialic acids from mucin glycans is the rate-limiting step in the total erosion of protective glycans (20). Continuing detailed characterization of the enzymatic machinery *C. perfringens* used for the hydrolysis of sialic acids from mucin glycans is critical to understanding mucin degradation and metabolism by this bacterium.

The genomes of most *C. perfringens* strains contain three separate genes encoding highly conserved GH33 CAZymes targeting sialic acid glycosidic linkages: the nonsecreted 43 kDa NanH, and the two secreted 77 kDa NanI and 129 kDa NanJ (20). NanH consists solely of the catalytic GH33 domain. NanI possess an additional N-terminal family 40 carbohydrate binding module (CBM). NanJ is highly multimodular comprising a central GH33 domain flanked on the N terminus by family 32 and 40 CBMs and on the C-terminal side by an unknown domain, X82 cohesin, and fibronectin type III-like domains (Fig. 1) (21, 22). CBM40 family members have been shown to bind sialic acid (23, 24), whereas polyspecific family 32 CBMs have demonstrated binding to other mucin-related glycans including galactose, lactose (25, 26), and *N*-acetyllactosamine (27). The multimodularity of *C. perfringens* CAZymes with CBMs, cohesin domains, and fibronectin type III-like domains has been proposed to promote binding and degradation of the complex glycans within the mucus layer (20). The X-ray crystallographic protein structure of the NanI catalytic domain was solved previously, as well as complexes with a known inhibitor and a catalytic intermediate. The enzyme adopts an overall β-propeller fold with an active site pocket for *exo*-sialidase activity (28) consistent with other bacterial (23, 29, 30, 31, 32), viral (33), and human (34) *exo*-sialidases. NanI displays the evolutionary retained elements that are consistent in the active site of sialidases (28, 35). The carboxylate group of a bound sialic acid interacts with the tri-arginine cluster (Arg^266^, Arg^555^, and Arg^615^ in NanI). A hydrophobic pocket (Phe^347^, Phe^353^, Phe^460^, Thr^345^ Ile^327^) accommodates the *N*-acetyl group of the sialic acid. A conserved aspartic acid (Asp^291^) functions as an acid/base catalyst (28). To date, structural information for the catalytic domains of NanH and NanJ is absent, while detailed functional comparisons of the three enzymes is limited. On the basis of sialidase activity in the extracellular supernatants of isogenic *C. perfringens* (strain CN3718) sialidase mutants, the three enzymes were reported to differ in their preference for sialic acid linkages (36). NanH was reported to be preferentially active on α-2,8 linkages (α-2,8 > α-2,3 > α-2,6), NanI on α-2,3 linkages (α-2,3 > α-2,6 > α-2,8), and NanJ on α-2,6-linkages (α-2,6 > α-2,8 > α-2,3). However, more detailed analyses of the isolated enzymes are needed to confirm their substrate preferences and to establish structure-function relationships conferring their preferences.

**Figure 1:**
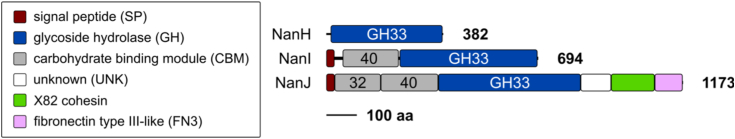
**Characterization of substrate scope of *Clostridium perfringens* sialidases.** The full-length sialidases of *C. perfringens* are displayed, with domain boundaries based on published data, or predicted by dbCAN (53) and InterProScan (54). Schematics are to scale, with polypeptide chain length indicated. Adapted from Low *et al.* (2021) (20). Note that this study examined recombinant constructs consisting of the catalytic GH33 domain only of each.

Given the expansion of GH33 encoding genes in *C. perfringens*, we hypothesized that the different versions of the enzymes may have different properties relevant to the processing of complex mucin *O*-glycan structures when deployed for nutrient harvesting. Here, we investigate more detailed structure-function relationships for the isolated GH33 catalytic domains of the three sialidases of *C. perfringens*. We refer to these constructs as NanH_GH33_, NanI_GH33_, and NanJ_GH33_. We found that these enzymes display distinct abilities to process variably modified forms of terminal sialic acids and differential specificities for unique stereochemical linkages of sialic acids. The data presented here have informed *in vivo* models for *C. perfringens* growth and metabolism of mucin *O*-glycans. We demonstrate the potential/proof of concept for inhibition of the breakdown of these glycans through treatment with a sialidase inhibitor. Together these data present a basis for future development of mitigation strategies *via* small molecule and substrate mimetics to treat *C. perfringens*-causative disease.

## Results

### Activities of the three *C. perfringens* GH33 enzymes

**Figure 2:**
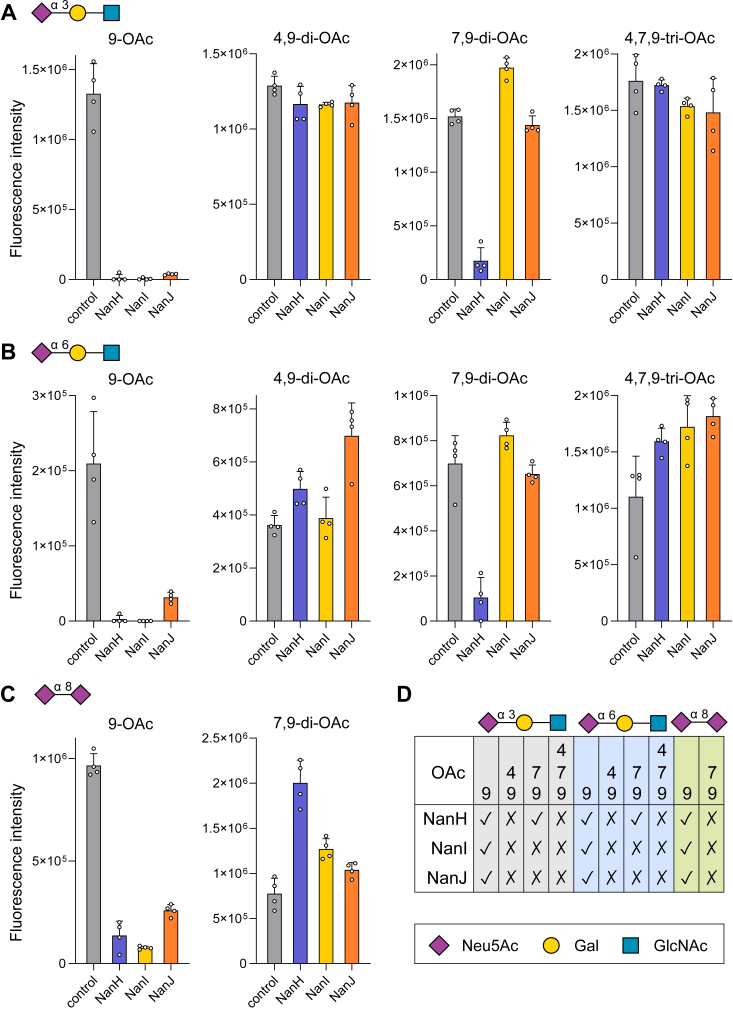
**Probing the substrate tolerance of *Clostridium perfringens* sialidases using the acetylated sialosides microarray.** The acetylated sialosides array was treated with NanH_GH33_, NanI_GH33_, and NanJ_GH33_ (20 μg/ml in pH 7.4 PBS buffer) at 37 °C for 1 h. Bovine coronavirus hemagglutinin-esterase (BCoV HE) was then used to probe the result of the sialidase treatment. Loss of binding signal after sialidase treatment suggests cleavage of the sialic acid. Microarray data for the untreated control and the NanH_GH33_, NanI_GH33_, and NanJ_GH33_ treated slides are shown for (*A*) acetylated 2,3-sialosides, (*B*) acetylated 2,6-sialosides, and (*C*) acetylated 2,8-sialosides. Data are shown as quadruplicate values, with the error bar representing standard deviation of the mean. *D*, summary of substrate tolerance of the sialidase on the acetylated sialosides.

The isolated GH33 domains of NanH, NanI, and NanJ (referred to as NanH_GH33_, NanI_GH33_, and NanJ_GH33_) were obtained by expression of the gene fragments encoding them in *E. coli* and subsequent protein purification by immobilized metal affinity chromatography. To provide initial insight into the substrate selectivities of the GH33 enzymes we used a previously described microarray method (37) to probe accommodation of sialic acid acetylation patterns in the context of how the sialic acid is linked to a preceding sugar moiety. The array of acetylated sialosides was treated with NanH_GH33_, NanI_GH33_, or NanJ_GH33_, followed by using bovine coronavirus hemagglutinin-esterase (BCoV HE) to probe the result of the sialidase treatment. Loss of BCoV HE binding after sialidase treatment indicates removal of the sialic acid. All three sialidases were found to be active on 9-*O*-Ac forms of α-2,3-sialyllactose, α-2,6-sialyllactose, and α-2,8-disialic acid (Fig. 2). The presence of a 4,9-*O*-Ac abrogated activity of all the enzymes. Only NanH_GH33_ was active on 3′- and 6′-sialyllactosamines with a 7,9-di-*O*-Ac Neu5Ac. None of the enzymes had activity on the 4,7,9-tri-*O*-Ac Neu5Ac sialosides. This suggests that a 4-*O*-Ac modification is inhibitory to all the enzymes while a 7-*O*-Ac modification is inhibitory to NanI_GH33_ and NanJ_GH33_.

**Figure 3:**
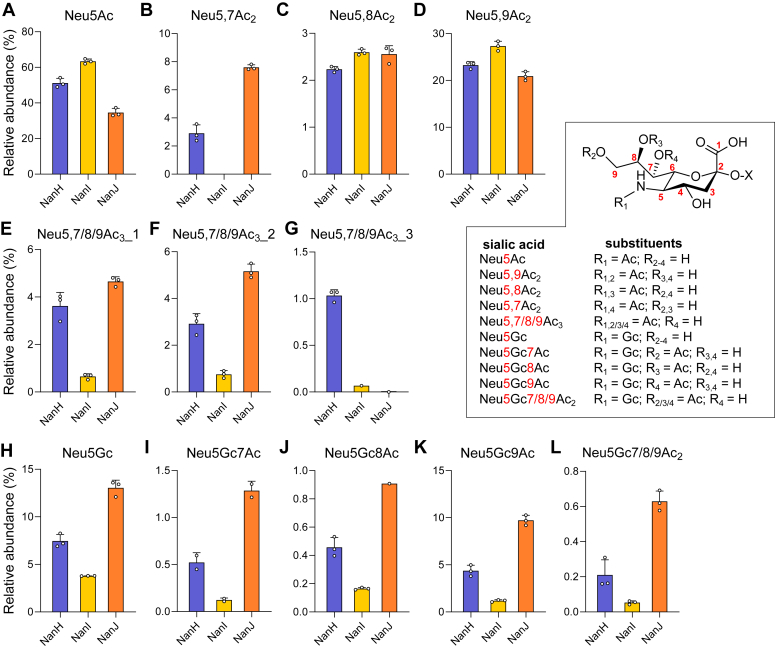
**Assessing the tolerance of *C. perfringens* sialidases against *O*-acetylated sialic acids.***A*–*L*, release of *O*-acetylated sialic acid species indicated in the panels by each enzyme. Bovine submaxillary mucin (BSM) was treated with NanI_GH33_, NanJ_GH33_, and NanH_GH33_ for 5.5 h after which liberated sialic acids were derivatized and analyzed by HPLC-MS. Activities of each sialidase were normalized based on their rate of hydrolyzing 4MU-Neu5Ac. Three replicates were assessed per sialidase, with error bars representing the standard deviation of the mean. In all instances, MS detector responses for each unique sialic acid were normalized to the total detectible sialic acid pool in each sample. Except for Neu5Ac, Neu5Gc, and Neu5,9Ac_2_, all assignments are putative albeit Neu5,7Ac2, Neu5,8Ac2 (and their Neu5Gc congeners) are consistent with their previously reported retention times and fragmentation patterns. In *E*-*G*, triacetylated substrates at four positions (5 and 7/8/9) were not confidently identified and are thus listed as Neu5,7/8/9_1, _2, and _3. Based on microarray data (Fig. 2), we propose what we have annotated as Neu5,7/8/9Ac3_3 is Neu5,7,9Ac3. Neu5Ac, *N*-acetylneuraminic acid; Neu5Gc, *N*-glycolylneuraminic acid; HPLC-MS, high-performance liquid chromatography coupled to mass spectrometry.

We further investigated the ability NanH_GH33_, NanI_GH33_, and NanJ_GH33_ to hydrolyze sialic acid variants by incubating bovine submaxillary mucin (BSM), a well-characterized source of *O*-acetylated sialic acids (38), with the enzymes and analyzing the released sialic acids by high-performance liquid chromatography coupled to mass spectrometry (Fig. 3). Neu5Ac was the most abundant sialic acid released. Diacetylated versions with additional 7-*O*-Ac, 8-*O*-Ac, and 9-*O*-Ac modifications, and triacetylated versions were also detected (Fig. 3, *A*–*G*). However, in the latter cases, the specific positions of the acetyl groups could not be resolved but were assumed to be the three possibilities involving pairs of 7-*O*-Ac, 8-*O*-Ac, and 9-*O*-Ac modifications in addition to the 5-*N*-Ac. NanI_GH33_ was unable to release Neu5,7Ac_2_ indicating clearly that the 7-*O*-Ac group inhibits the hydrolysis these terminal sialic acid variants. NanJ_GH33_ was able to release this sialic acid variant, but was inactive on Neu5,7,9Ac_3_ in the arrays, suggesting that accommodation of the 7-*O*-Ac group by NanJ_GH33_ may be context dependent. Indeed, one of the tri-acetylated variants was resistant to both NanI_GH33_ and NanJ_GH33_, like the Neu5,7,9Ac_3_ on the arrays, and we thus propose what we have annotated as Neu5,7/8/9Ac_3__3 is Neu5,7,9Ac_3_. In general, NanI_GH33_ displayed comparatively poor ability to release triacetylated sialic acid variants from BSM, whereas NanJ_GH33_ was active on the two versions that are assumed to be Neu5,8,9Ac_3_ and Neu5,7,8Ac_3_, and NanH_GH33_ was active on all of them.

All of the enzymes displayed some capacity to release from BSM Neu5Gc and acetylated variants thereof, though we were unable to assign absolute structures to these sugars and they are thus inferred (Fig. 3, *H*–*L*). Based on the relative abundances of the released Neu5Gc variants, and comparison across the three enzymes, it appears that NanI_GH33_ is the least able at releasing Neu5Gc, whereas NanJ_GH33_ may be marginally better than NanH_GH33_.

Overall, these results indicate that the acetylation of sialic acid plays a strong role in determining whether this terminal residue is a substrate for the *C. perfringens* sialidases, with NanH_GH33_ being the most tolerant, followed by NanJ_GH33_, and finally NanI_GH33_ being the least tolerant of acetyl groups in particular positions and combinations. To probe the structural features underlying this selectivity, we used X-ray crystallography to determine the structures of the three enzymes in complex with various ligands.

### Structural analysis of the *C. perfringens* sialidases

**Figure 4:**
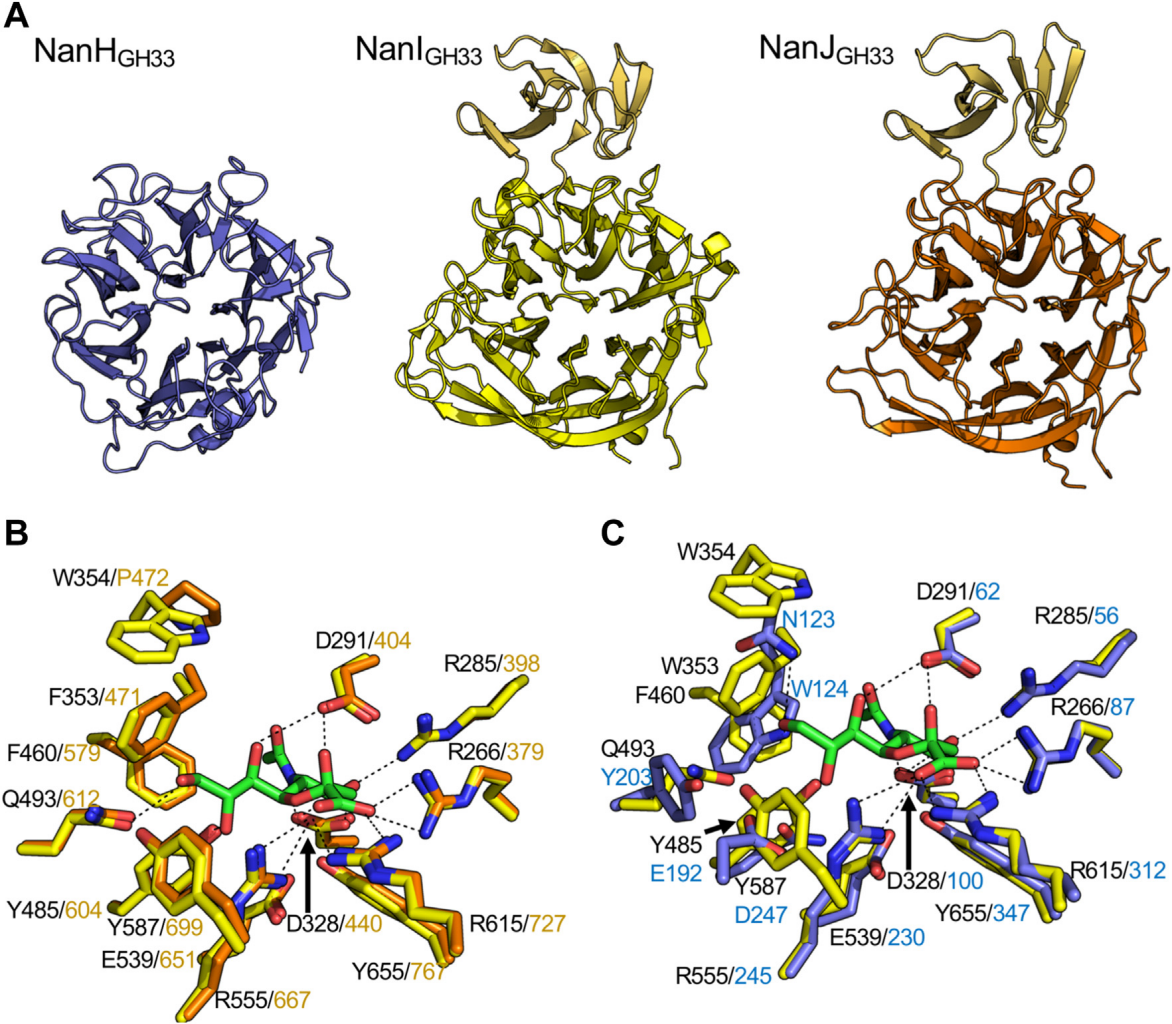
**Structures of NanI, NanJ**_**GH33**_**, and NanH**_**GH33**_. *A*, a comparison of the catalytic domains of NanJ_GH33_ (*orange*), NanH_GH33_ (*blue*), and NanI (*yellow*, PDB code: 2VK5). The insertion loop in NanJ_GH33_ and NanI is highlighted in *light orange*/*yellow*. *B*, Neu5Ac complexed in the −1 binding site of NanJ_GH33_ and NanI (PDB code: 2BF6). *C*, Neu5Ac complexed in the −1 binding site of NanH_GH33_ and NanI (PDB code: 2BF6). The distance cutoff for H-bonds, shown as *dashed black lines*, was chosen as 3.3 Å. Neu5Ac, *N*-acetylneuraminic acid; PDB, Protein Data Bank.

A comparison of the amino acid sequence of the three sialidases indicates that NanI_GH33_ and NanJ_GH33_ share 60% amino acid sequence identity, compared with a relatively low 29% and 27% amino acid sequence identity with NanH_GH33_, respectively. We solved the unliganded structures of NanH_GH33_ and NanJ_GH33_ and compared them with the previously determined structure of NanI_GH33_ (Protein Data Bank (PDB) ID 2VK5 (28) (Fig. 4). The structures of NanH_GH33_ and NanJ_GH33_ display the characteristic 6-bladed β-propeller fold seen in *exo*-acting sialidase GH33 domains (39). The structures of NanJ_GH33_ and NanI_GH33_ were very similar with an RMSD of 1.1 Å and with both proteins containing the same β-sheet domain insertion within the catalytic domain (between residues 477–546 in NanJ_GH33_ and 361–427 in NanI_GH33_). The inserted domain is not present in NanH_GH33_ and the RMSD of NanH_GH33_ with the other two structures is 1.7 Å. The inserted domain is not within the proximity of the catalytic site of the sialidases, and therefore likely does not directly impact the activity of the sialidase.

The structures of NanH_GH33_ and NanJ_GH33_ in complex with the Neu5Ac (Fig. S1) compared to the existing structure of NanI in complex with Neu5Ac (PDB ID 6BF6) revealed the properties of the −1 subsites in these enzymes. The amino acid side chains forming the active site pockets of NanJ_GH33_ and NanI_GH33_ were identical, though a notable position in a second shell of active site residues showed a tryptophan (W354 in NanI_GH33_) *versus* proline (P472 in NanJ_GH33_) difference (Fig. 4*B*). Similarly, the amino acid sidechains in the active sites of NanH_GH33_ and NanI_GH33_ that interact with the Neu5Ac ring structure, but not the glycerol substituent, were also identical (Fig. 4*C*). Further, this specific region of the active site is highly conserved across all three enzymes and prevents recognition of 4-*O*-acetylated sialic acid variants *via* an arginine residue (R285 in NanI_GH33_, R398 in NanJ_GH33_, and R56 in NanH_GH33_) that blocks accommodation of extensions on C4. NanH_GH33_, however, has a considerably different organization in the active site region that interacts with the glycerol moiety, suggesting this may contribute to the ability of this enzyme to recognize additional acetylated variants of sialic acid.

**Figure 5:**
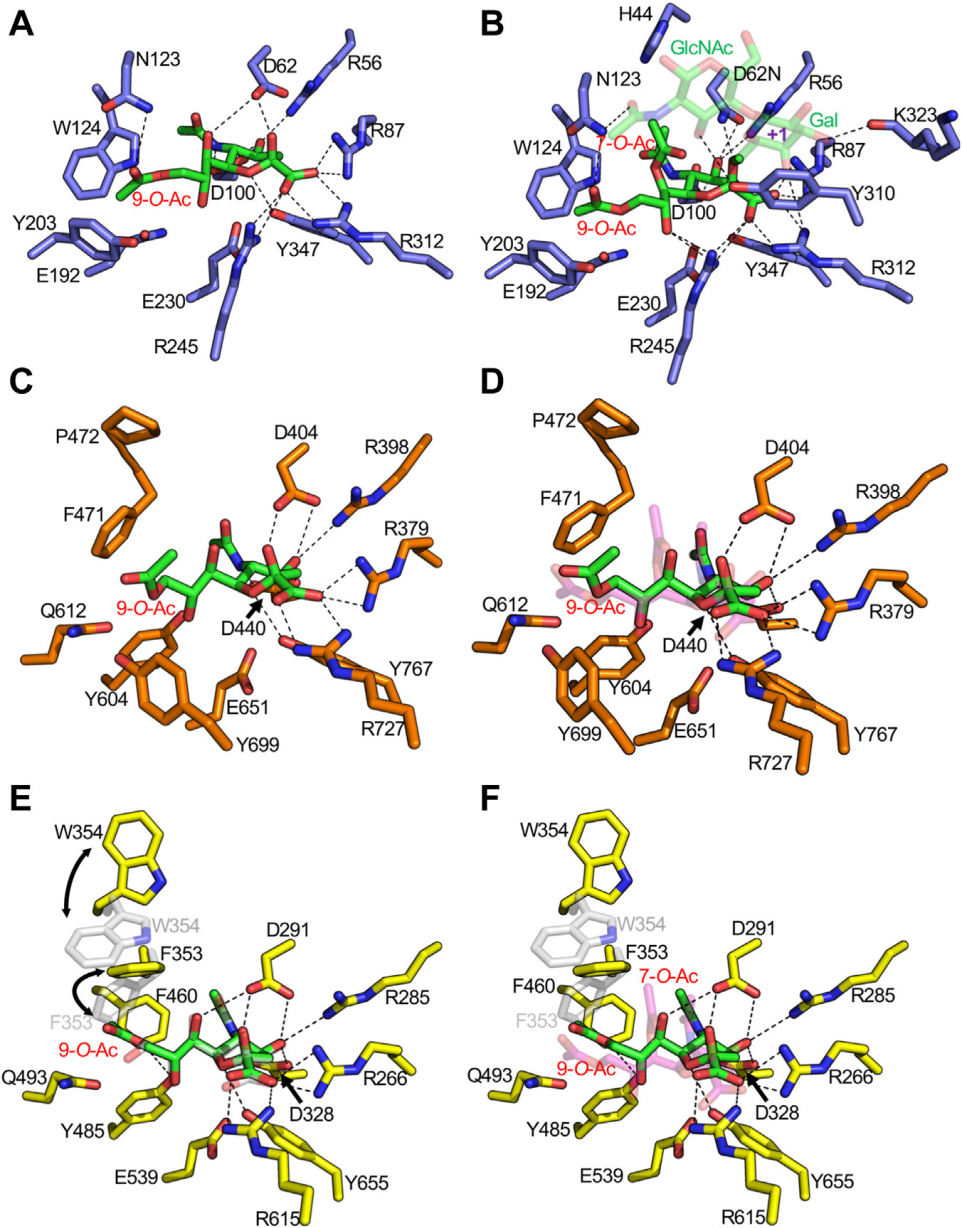
**Structures of NanI**_**GH33**_**, NanJ**_**GH33**_**, and NanH**_**GH33**_**in complex with sialic acid variants.***A*, NanH_GH33_ in complex with Neu5,9Ac. *B*, NanH_GH33_ D62N in complex with SAc_3_LacNAc. The *N*-acetyllactosamine (LacNAc) moiety is shown as transparent to focus on the Neu5,7,9Ac residue in the −1 subsite. The location of the +1 subsite is labeled in *purple*. *C*, NanJ_GH33_ in complex with Neu5,9Ac. *D*, NanJ_GH33_ in complex with Neu5,9Ac overlapped with the Neu5,7,9Ac residue from SAc_3_LacNAc in the NanH_GH33_ D62N complex; Neu5,7,9Ac is shown as transparent *magenta sticks*. *E*, NanI_GH33_ in complex with Neu5,9Ac (*yellow* and *green*) overlapped with the NanI Neu5Ac complex (*transparent gray*, PDB ID 2BF6). *Arrows* highlight the conformational differences of specific sidechains. *F*, NanI_GH33_ in complex with Neu5,9Ac (*yellow* and *green*) with conformationally flexible sidechains in the NanI Neu5Ac complex (transparent *gray*, PDB ID 2BF6) overlapped with the Neu5,7,9Ac residue from SAc_3_LacNAc in the NanH_GH33_ D62N complex; Neu5,7,9Ac is shown as *transparent magenta sticks*. The distance cutoff for H-bonds, shown as *dashed black lines*, was chosen as 3.3 Å. Neu5Ac, *N*-acetylneuraminic acid; PDB, Protein Data Bank.

We explored this hypothesis by determining the structure of NanH_GH33_ with bound Neu5,9Ac_2_ and an inactive D62N mutant in complex with a trisaccharide comprising *N*-acetyllactosamine terminating in α-2-6-linked Neu5,7,9Ac_3_ (referred to as SAc_3_LacNAc) (Fig. 5, *A* and *B*, Fig. S1). Neither structure showed any significant perturbation of active site residues relative to either the unliganded or Neu5Ac complex. The 9-*O*-Ac group of both ligands project into a preformed pocket formed largely by N123, W124, and Y203. The 7-*O*-Ac group of SAc_3_LacNAc also projects up into a preexisting space in the active site. This reveals how these modifications are accommodated by the active site of NanH_GH33_, which is notable as they both involve the residues in the active site region of NanH_GH33_ that are not conserved in NanI_GH33_ and NanJ_GH33_. In NanI_GH33_ and NanJ_GH33_, the 9-*O*-Ac binding site is apparently blocked off by phenylalanine residues (F353 and F471 in NanI_GH33_ and NanJ_GH33_, respectively) (Fig. 5, *C*–*F*). In NanH_GH33_, the 7-*O*-Ac group of SAc_3_LacNAc nestles against N123, which is spatially occupied in NanI_GH33_ and NanJ_GH33_ by the larger phenylalanine residue that also blocks the 9-*O*-Ac pocket.

The positions of the active site residues in the NanJ_GH33_ Neu5,9Ac_2_ complex remained essentially identical to those in the NanJ_GH33_ Neu5Ac complex, continuing to suggest a relatively rigid active site (Fig 5*C*, Fig. S1). The 9-*O*-Ac group was accommodated by this group being orientated up and away from the active site, rather than continuing the trajectory of the glycerol chain into the a 9-*O*-Ac binding pocket as in NanH_GH33_ (Fig. 5*D*). With knowledge of how the 7-*O*-Ac group of SAc_3_LacNAc is accommodated by NanH_GH33_, there is nothing to suggest in the NanJ_GH33_ Neu5Ac complex that NanJ_GH33_ would be unable to recognize a 7-*O*-Ac modification in very similar conformation. Indeed, NanJ_GH33_ was able to release Neu5,7Ac_2_ from BSM. The presence of the 9-*O*-Ac group, however, and how it is positioned in the NanJ_GH33_ active site appears to change the context of other acetyl groups. The conformation of the 9-*O*-Ac group in the NanJ_GH33_ complex would be anticipated to clash with the conformation of a 7-*O*-Ac group if positioned in a manner that would allow accommodation in the NanJ_GH33_ active site. We propose this as a plausible structural explanation for why NanJ_GH33_ was inactive on Neu5,7,9Ac_3_ on the arrays but is active on the individual Neu5,7Ac_2_ and Neu5,9Ac_2_ variants.

In contrast to NanH_GH33_ and NanJ_GH33_ binding Neu5,9Ac_2_, the binding of this monosaccharide to NanI_GH33_ caused a structural rearrangement in the active site. The side chain of W354 swivels approximately 180^o^, which in turn allows the sidechain of F353 to move ∼90^o^ clockwise and occupy the vacated space (Fig. 5*E*, Fig. S1). This opens up the 9-*O*-Ac binding pocket that was otherwise blocked by F353 allowing Neu5,9Ac_2_ to be bound by NanI_GH33_ in a conformation similar to that observed in the NanH_GH33_ complex. This structural rearrangement is not possible in NanJ_GH33_ due to the presence of P472, which is locked in place and prevents significant movement of the F471 sidechain, thus requiring the structural rearrangement of the Neu5,9Ac_2_ instead. The inability of NanI_GH33_ to recognize 7-*O*-Ac modified sialic acid in any capacity is likely due to the F353/W354 combination (Fig. 5*E*). When the 9-*O*-Ac pocket is unoccupied it appears that W354 blocks the region that would otherwise accommodate a 7-*O*-Ac (this position is the smaller P472 residue in NanJ_GH33_). Though W354 moves when binding a ligand with a 9-*O*-Ac, the rotation of F353 would cause its sidechain to block 7-O-Ac binding.

**Figure 6:**
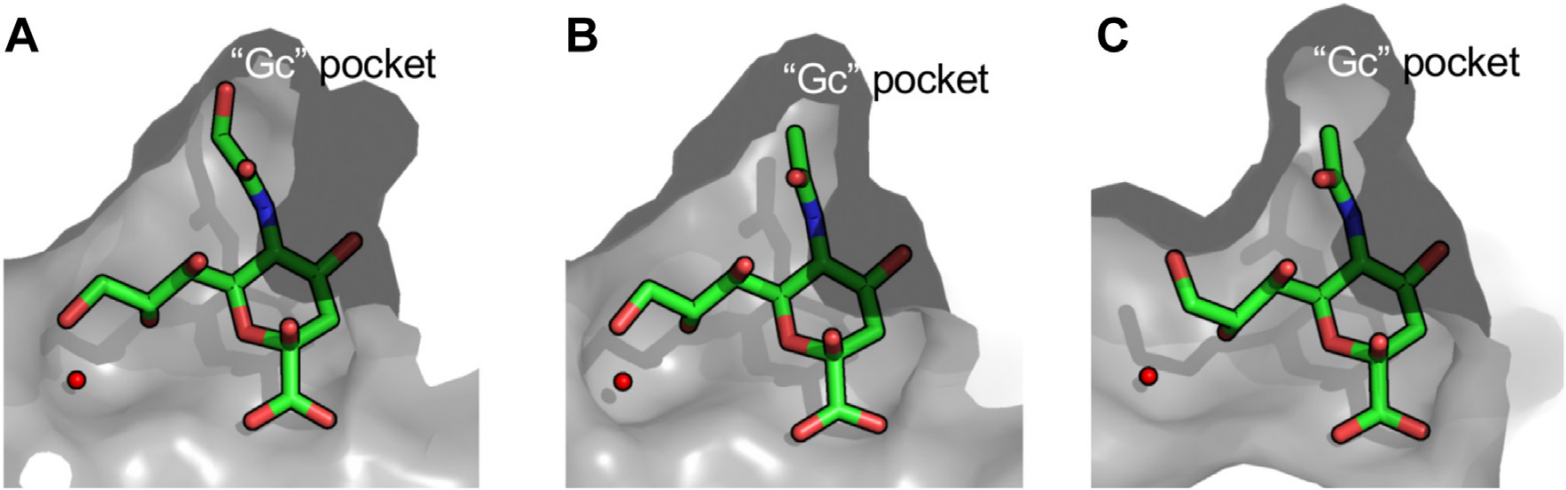
**NanI**_**GH33**_**in complex with Neu5Gc reveals a “Gc” binding pocket.***A*, solvent accessible surface of the crystal structure of NanI_GH33_ in complex with Neu5Gc. *B*, solvent accessible surface of the −1 subsite in the crystal structure of NanJ_GH33_ Neu5Ac complex and (*C*) the crystal structure of NanH_GH33_ Neu5Ac complex. The pocket accommodating the glycerol group of Neu5Gc is highlighted in each panel. The *red sphere* in each panel is the conserved water molecule that occupies a putative 8-O-Ac binding pocket. Neu5Ac, *N*-acetylneuraminic acid; Neu5Gc, *N*-glycolylneuraminic acid.

We did not have simple ligands or substrates with 8-*O*-Ac modifications and thus we were unable to directly probe the structural basis of how this group is accommodated by the three enzymes. The structures we obtained, however, revealed conserved water in a pocket beneath O8 of the glycerol moiety (Fig. 6). This space is likely sufficient in all of the enzymes to accommodate the acetyl group of an 8-*O*-Ac modification.

Generally, the dominant form of sialic acid in *O*-glycans is Neu5Ac, and is thus the most likely substrate of the *C. perfringens* sialidases. However, Neu5Gc (and its alternately acetylated species) is a naturally occurring variant of sialic acid that is present in BSM and was released to some extent by all of the enzymes, though low abundance made it difficult to confidently assign the structures of the acetylated variants. We were able to generate a complex of NanI_GH33_ with Neu5Gc, which highlighted a “Gc pocket” that can accommodate the additional O11 hydroxyl group of Neu5Gc (Fig. 6*A*). This pocket also appears to be present in NanJ_GH33_ and NanH_GH33_, which is consistent with their ability to release this monosaccharide (Fig. 6, *B* and *C*).

### Linkage specificity of the *C. perfringens* sialidases

**Figure 7:**
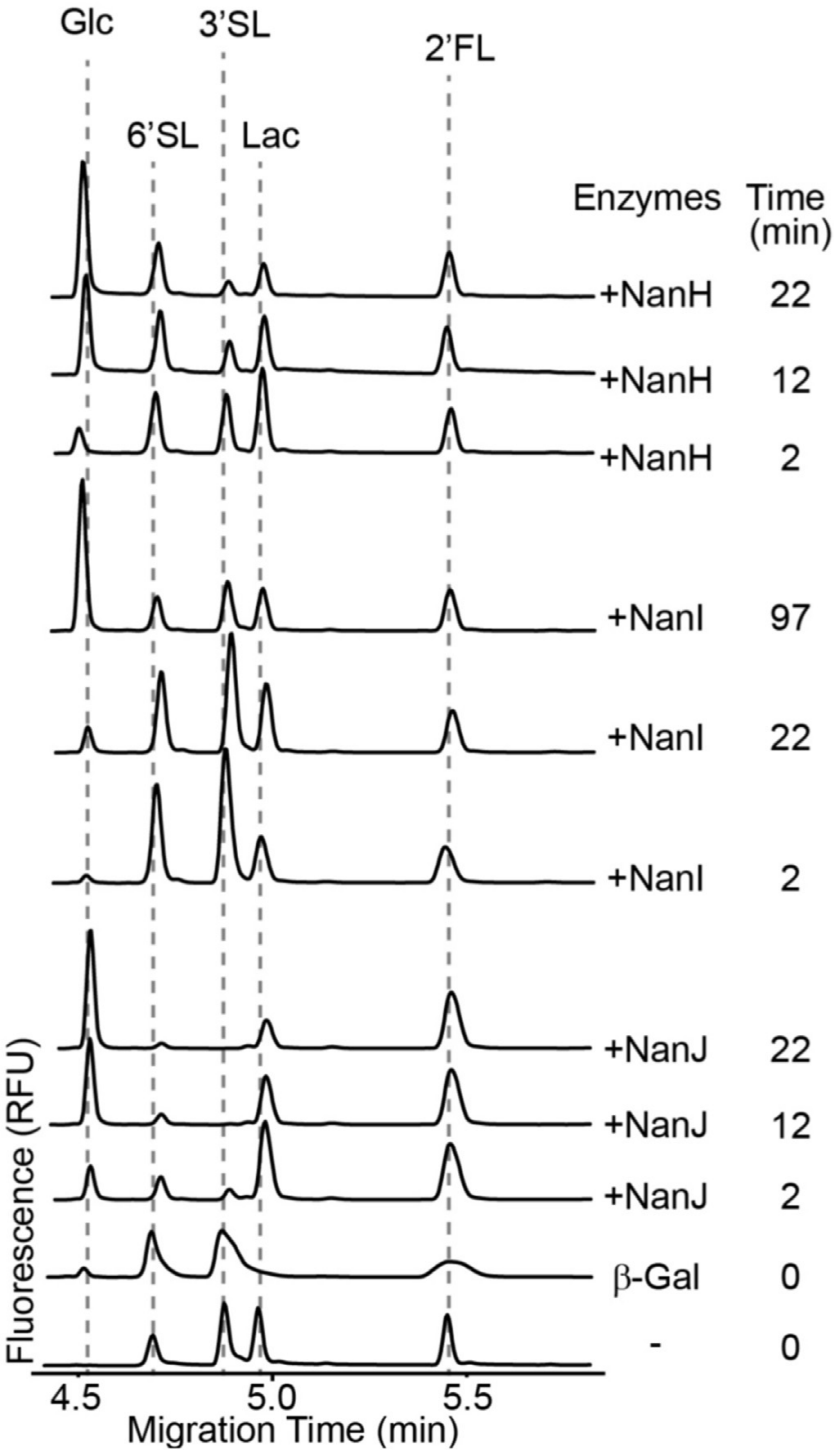
**CE-LIF-based substrate reduction assays reveal activity against α2,3/6-linked sialosides for NanH**_**GH33**_**, NanI**_**GH33**_**, and NanJ**_**GH33**_. Mixtures of fluorogenically labeled 3’/6′-SL contain lactose (Lac) which was removed from the assay mixture by pretreatment with β-galactosidase to yield labeled glucose (Glc). Neuraminidases were added and immediately analyzed by CE-LIF, the first time point reflecting the 2 min capillary preconditioning prior to sample injection. CE-LIF, capillary electrophoresis with laser induced flourescence.

While the selectivity of the three *C. perfringens* sialidases has been hinted at previously, we sought to take a more quantitative *in vitro* approach to examining the selectivity of the enzymes for the most common terminal sialic acid linkages. We used capillary electrophoresis with laser induced flourescence for detection (CE-LIF) to monitor 3′-sialyllactose (3′-SL) and 6′-sialyllactose (6′-SL) hydrolysis in a competition assay with equal amounts of each substrate over relatively short time periods (Fig. 7). Reactions included a comparatively high concentration of β-galactosidase to remove the lactose product and prevent the potent trans-sialidase activity of the enzymes. When provided with both substrates at the same time, NanH_GH33_ hydrolyzed 3′-SL but did not show any significant capacity to hydrolyze 6′-SL (Fig. 7). However, in the absence of a competing α-2,3-linked sialic acid substrate NanH_GH33_ was clearly able to hydrolyze α-2,6-linked sialic acid in the microarray analysis (Fig. 2*B*). NanI_GH33_ showed roughly equal rates of 3′-SL and 6′-SL depletion, revealing little to no selectivity (Fig. 7). NanJ_GH33_ processed both substrates very rapidly, again suggesting limited selectivity for α-2,3- *versus* α-2,6-linked sialic acid (Fig. 7) when both substrates are provided simultaneously.

### Inhibition of the *C. perfringens* sialidases

**Figure 8:**
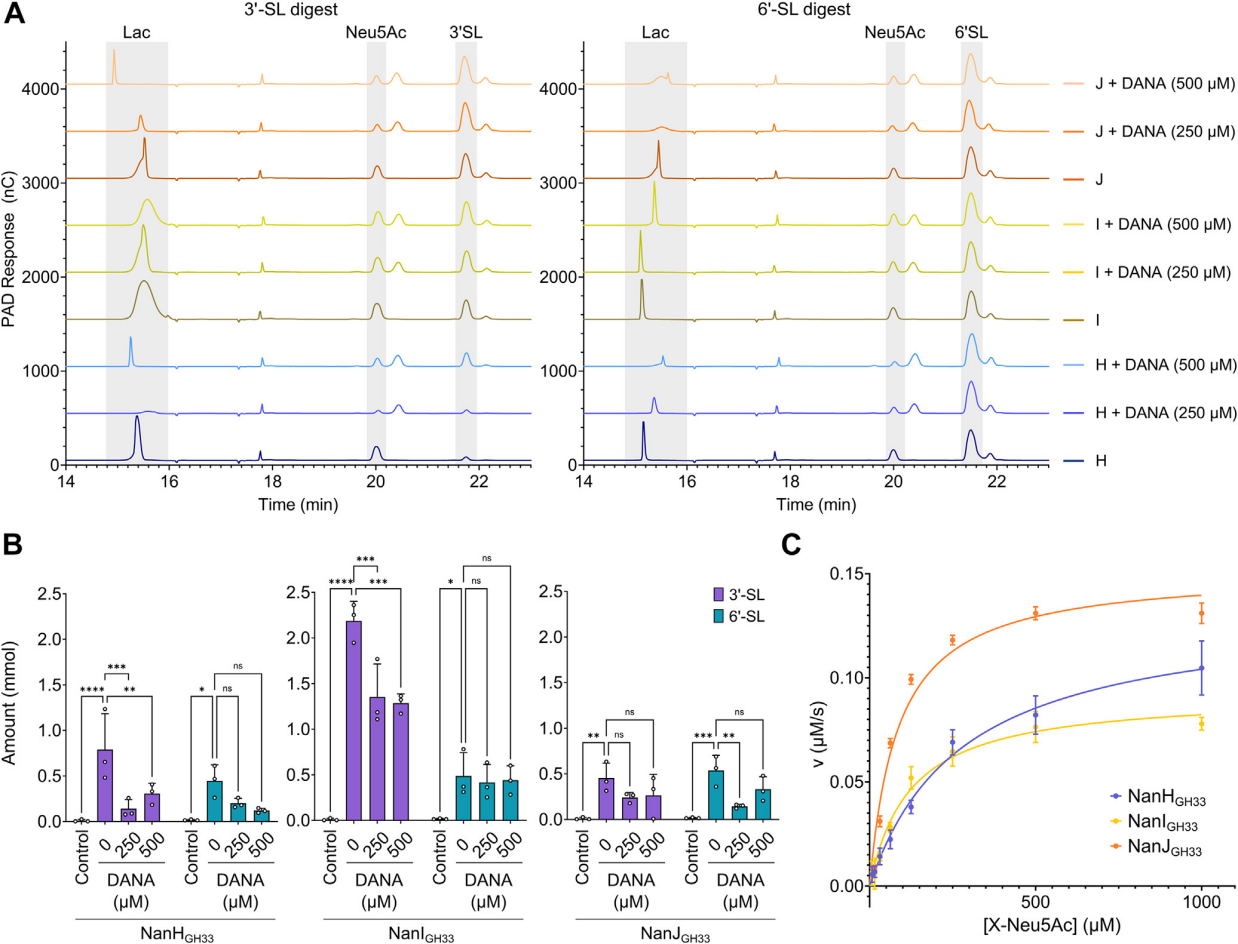
**Enzymatic hydrolysis of sialyllactose and X-Neu5Ac substrates.***A*, enzymatic digests of 3′-sialyllactose and 6′-sialyllactose substrates by the sialidases were analyzed by HPAEC-PAD in triplicate, with one replicate trace shown. *B*, released Lac, as analyzed by HPAEC-PAD, was quantified using Chromeleon software (Thermo Fisher Scientific) and the known concentration series of standards as outlined. Amounts of Lac disaccharide are shown as averages of triplicate HPAEC-PAD injections, and error bars represent the standard deviation of the mean. Statistical significance is indicated by asterisks (∗ = *p* < 0.05, ∗∗ = *p* < 0.005, ∗∗∗ = *p* < 0.0005, ∗∗∗∗ = *p* < 0.0001), as calculated using a 2-way ANOVA. *C*, the initial rates of X-Neu5Ac substrate hydrolysis by the GH33 domains were obtained and fit to the Michaelis-Menten equation. Data were obtained in triplicate, and error bars represent the standard error of the mean. Neu5Ac, *N*-acetylneuraminic acid; X-Neu5Ac, 5-bromo-4-chloro-3-indolyl α-D-N-acetylneuraminic acid; HPAEC-PAD, high-performance anion-exchange chromatography with pulsed amperometric detection.

There exists numerous sialidase inhibitors with previously demonstrated activity against human (40) and viral (33, 41, 42) neuraminidases. The inhibition of *C. perfringens* sialidases has been previously investigated, however, limited by the use of cell culture supernatants (36) or unspecified enzyme (43). Here, we assessed known neuraminidase-active compounds, including inhibitors of human and influenza neuraminidases, for their potential inhibition of the three purified *C. perfringens* GH33 enzymes: *N*-acetyl-2,3-dehydro-2-deoxyneuraminic acid (DANA), (3S, 4S, 5R, 6R)-6-(acetylamino)-4,5-dihydroxy-3-piperidinecarboxylic acid (siastatin B), (1S,2S,3R,4R)-3-[(1S)-1-(acetylamino)-2-ethylbutyl]-4-[(aminoiminomethyl)amino]-2-hydroxycyclopentanecarboxylic acid (peramivir), 4-guanidino-2,4-dideoxy-2,3-dehydro-*N*-acetylneuraminic acid (zanamivir), and (3R,4R,5S)-4-acetamido-5-amino-3-(1-ethylpropoxy)-1-cyclohexene-1-carboxylic acid ethyl ester (oseltamivir). Initially, DANA was used to investigate the potential for sialidase inhibitors to protect against hydrolysis of sialyllactose substrates (Fig. 8, *A* and *B*). Indeed, DANA was found to inhibit the activity of the three GH33 sialidases and reduced the amount of lactose released by NanH_GH33_, NanI_GH33_, and NanJ_GH33_ from 3′- and 6′-SL. To better quantify inhibition, we used the chromogenic substrate X-Neu5Ac, beginning with determination of the kinetic parameters for all three sialidases at each of the enzymes pH optimum (Fig. S2). The resulting data were fit to the standard Michaelis-Menten model (Fig. 8*C* and Fig. S3). NanJ_GH33_ was found to be the most kinetically efficient at hydrolysis of X-Neu5Ac, with approximately 2-fold and 3.6-fold the k_cat_/K_m_ of NanI_GH33_ and NanH_GH33_, respectively (Table 1). Enzyme inhibition kinetics of the five inhibitors tested revealed that zanamivir and oseltamivir did not display any detectable inhibition of the three GH33 domains at the concentrations tested (up to 1 mM) (Table 1). Peramivir was found to inhibit NanJ_GH33_ weakly with a K_i_ of 104.0 ± 28.4 μM but it did not inhibit NanH_GH33_ or NanI_GH33_. DANA and siastatin B were found to inhibit all three GH33s. DANA was equally potent against NanI_GH33_ and NanJ_GH33_ with K_i_s of 3.1 ± 0.5 μM and 3.2 ± 0.3 μM, respectively; it inhibited NanH_GH33_ to a lesser extent with a K_i_ of 33.9 ± 4.4 μM. Siastatin B was a less potent inhibitor of DANA, with higher K_i_ values against NanH_GH33_, NanI_GH33_, and NanJ_GH33_, of 125.0 ± 16.2 μM, 10.7 ± 2.0 μM, and 13.3 ± 1.6 μM, respectively. Both DANA and siastatin B were slightly weaker inhibitors of NanH_GH33_ than NanI_GH33_ and NanJ_GH33_. All inhibition data were consistent with a competitive mode of inhibition, as one would predict for sialic acid derivatives.

**Table 1:** Kinetic parameters for uninhibited and inhibited hydrolysis of X-Neu5Ac

Kinetic constant	NanH	NanI	NanJ
V_max_ (μM/s)	0.13 ± 0.01	0.09 ± 0.01	0.13 ± 0.01
K_m_ (μM)	280.0 ± 62.7	123.0 ± 25.4	87.0 ± 10.7
k_cat_ (1/s)	13.3 ± 1.2	9.2 ± 0.6	15.3 ± 0.6
k_cat_/K_m_	0.05 ± 0.01	0.08 ± 0.02	0.18 ± 0.02
K_i_ (μM)			
DANA	33.9 ± 4.4	3.1 ± 0.5	3.2 ± 0.3
Siastatin B	125.0 ± 16.2	10.7 ± 2.0	13.3 ± 1.6
Peramivir	ND	ND	104.0 ± 28.4
Zanamivir	ND	ND	ND
Oseltamivir	ND	ND	ND

Standard error is shown. ND indicates inhibition too weak to be detected or quantified.

**Figure 9:**
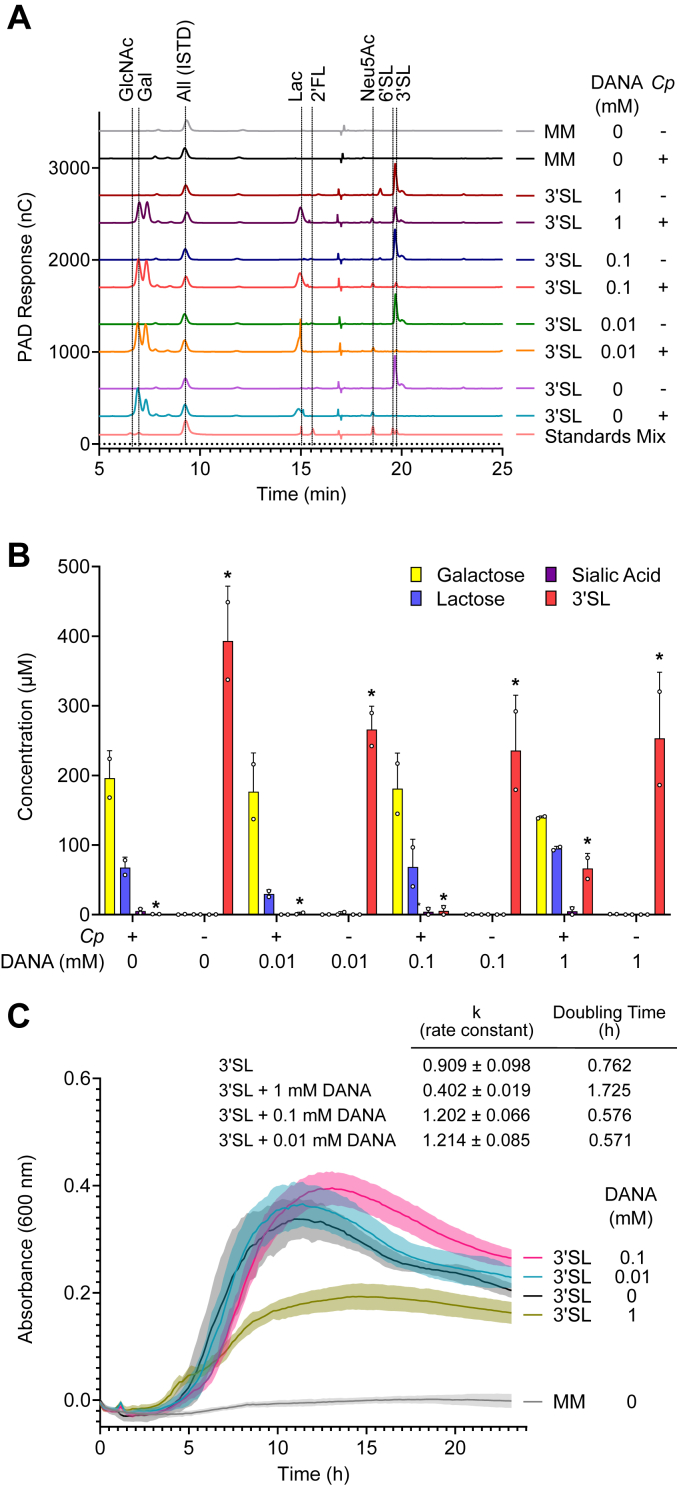
**Detection of carbohydrates remaining in *Clostridium perfringens* culture supernatants.***A*, representative HPAEC-PAD traces of filtered cell-free supernatants from 24 h cultures or negative controls containing 0.5% 3′-sialyllactose (3′-SL) and indicated concentrations of DANA. A dilution series of a standards mix of known milk oligosaccharides and monosaccharides was run in parallel with the samples to allow for quantification with an allose internal standard (ISTD) to account for variation in instrument injection volumes. Peaks from standards in the mix are indicated. *B*, quantification of known carbohydrates from *C. perfringens* supernatants. Error bars show the standard deviation of the mean (SD; n = 2 biological replicates) and asterisks indicate significant (*p* < 0.05) differences relative to the leftmost treatment group (+*C. perfringens*, - DANA) using a 2-way ANOVA. *C*, *Clostridium perfringens* growth curves in the presence of increasing concentrations of DANA neuraminidase inhibitor using 3′-SL as a substrate, with optical density monitored by absorbance quantitation at 600 nm. Averages of six growth curves are shown, with areas representing the standard deviation (SD). MM indicates minimalized medium with no carbohydrate. Bacterial growth kinetics were calculated based on exponential growth (GraphPad Prism), and rate constants and doubling times are listed for each growth condition. DANA, *N*-acetyl-2,3-dehydro-2-deoxyneuraminic acid; HPAEC-PAD, high-performance anion-exchange chromatography with pulsed amperometric detection.

To investigate if sialidase inhibitors would demonstrate potential in treatment of *C. perfringens* colonization and infection we used a *C. perfringens* growth assay and DANA as a model inhibitor, as it was found to effectively inhibit the three GH33 domains of NanH, NanI, and NanJ. DANA was found to protect against *C. perfringens* consumption of 3′-SL, as assessed by high-performance anion-exchange chromatography-pulsed amperometric (HPAEC-PAD) quantification (Fig. 9, *A* and *B*). Furthermore, DANA was also found to reduce the growth of *C. perfringens* in the presence of 3′SL (Fig. 9*C*) but not with glucose as the sole carbohydrate source (data not shown). However, a high concentration of DANA (1 mM) was required to demonstrate a clear effect. This may be due to the relatively low potency of DANA against the sialidases in the direct enzyme assays as well as the complexity of potential cellular interplay of all three sialidases at once.

## Discussion

The genomes of most *C. perfringens* strains encode three GH33 isozymes, suggesting the potential for diversified, rather than redundant, function. The observation that these isozymes only share 30 to 60% amino acid sequence identity and different modular architectures further supports this concept. Here, we have specifically probed this idea through structural and functional analysis of the GH33 domains from these enzymes. As a cohort of enzymes they possess broad specificity and provide the machinery to process multiple different variants of terminal sialic acid attached *via* different linkages.

NanJ_GH33_ was more efficient than NanI_GH33_ at hydrolyzing Neu5Gc and could accommodate 7-O-Ac modification, but the latter only when 9-O-acetylation was absent. NanI_GH33_ was most stringent being entirely unable to accommodate 7-O-Ac modification and was comparatively poor at hydrolyzing Neu5Gc variants. Neither enzyme could hydrolyze sialic acid with 4-*O*-Ac modifications. These two enzymes, which are predicted to be extracellular, are therefore somewhat redundant in their apparent specificity. NanI_GH33_ also proved to be the less efficient enzyme on a synthetic chromogenic substrate.

NanH_GH33_ proved to be the enzyme with the broadest activity with respect to accommodations of sialic acid variants, being able to process all common variants of sialic acid, except those with 4-*O*-Ac modifications, and did so with little obvious preference. The broad selectivity of NanH_GH33_ for these variants in comparison to NanI_GH33_ and NanJ_GH33_ appears to come from differences in the region responsible for recognition of 9-*O*-Ac and 7-*O*-Ac modifications. The NanH_GH33_ active site is preformed and suited to accommodate these modifications without any obvious conformational changes to the protein or substrate, unlike the requirements of NanI_GH33_ and NanJ_GH33_.

**Figure 10:**
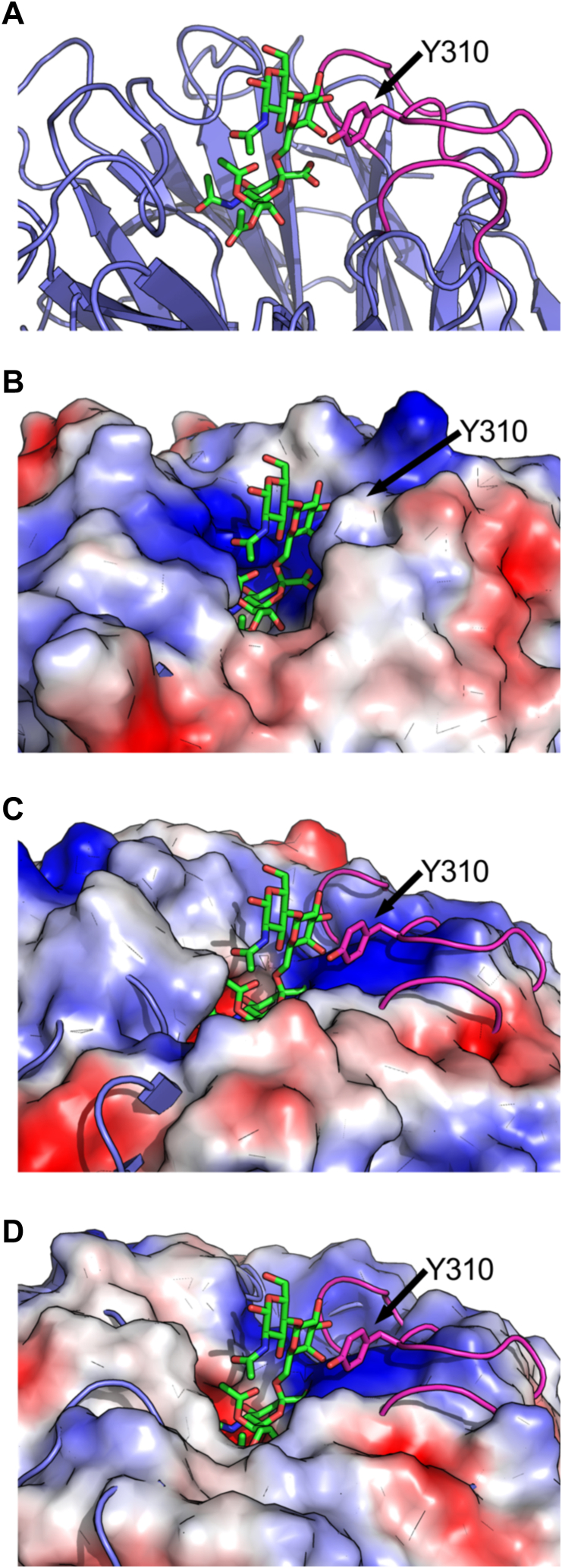
**NanH has an additional +1 subsite.***A*, secondary structure of NanH_GH33_ (*blue*) in complex with SAc_3_LacNAc (*green sticks*) showing the three extended loops surrounding the active site (*pink*). Y310 that packs against the galactose in the +1 subsite is shown as *sticks*. *B*, surface of NanH_GH33_ showing the packing of the extended glycan against the extended surface of Y310 and the loops. *C*, NanI_GH33_ (shown as a surface) overlapped with NanH_GH33_ in complex with SAc_3_LacNAc (as shown in panel *A*). *D*, NanJ_GH33_ (shown as a surface) overlapped with NanH_GH33_ in complex with SAc_3_LacNAc (as shown in panel *A*). Surfaces are shown as color ramped from *red* to *white* to *blue* as by their predicted negative to neutral to positive charge.

The most notable difference in NanH is in the loop structures surrounding the active site. The catalytic site of GH33 sialidases is in the cavity formed at the center of the 6-blades of the β-propellor fold. The loops of the propellers form a rim around the cavity. In NanH_GH33_ loops on 3-blades forming the rim of one side of the cavity are extended, thus deepening the active site (Fig. 10, *A* and *B*). The center blade of the three has a solvent exposed tyrosine residue, Y310, that projects into the active site. In the SAc_3_LacNAc Michaelis complex this tyrosine packs against the galactose to which the sialic acid is α-2,6-linked, providing additional interactions in a +1 subsite. In contrast, these loops are relatively minimal in NanI_GH33_ and NanJ_GH33_, creating a somewhat shallow sialic acid binding site (Fig. 10, *C* and *D*). This lack of a +1 binding site in either NanI_GH33_ or NanJ_GH33_ supports a general absence of selectivity for α-2,3- or α-2,6- linkages. In the case of the NanH_GH33_ SAc_3_LacNAc, however, the O4 of the galactose residue in the +1 subsite is oriented toward the aromatic ring of Y310, possibly making this less optimal interaction. Presumably, a similar interaction involving Y310 occurs when NanH_GH33_ binds α-2,3-linked sialoglycosides but in an as yet unknown but more favorable relative orientation of the galactose in the +1 subsite. Other GH33 sialidases have a +1 subsite with an aromatic amino acid sidechain, like NanH_GH33_ [*e.g.* TcTs from *Trypanosoma cruzi* (44) and NanC from *Streptococcus pneumoniae* (31)], whereas others have shallow active site like NanI_GH33_ and NanJ_GH33_ [*e.g.* PG0352 from *Porphyromonas gingivalis* (45)]. However, current data are insufficient to provide a clear link between the presence of a +1 subsite and linkage or aglycon specificity, thus further work is required to provide an explanation.

Overall, consistent with our hypothesis, the three *C. perfringens* sialidases do display some diversification of their substrate selectivity; however, there are more redundancies than differences, and it does not adequately justify the expansion of these enzymes in this bacterium. The most profound difference in the enzymes regards their likely modes of deployment. NanI and NanJ are both modular enzymes (Fig. 1), including modules characterized as having carbohydrate binding activity (22). However, NanI is predicted to be fully secreted while NanJ has a cohesin module that participates in the formation of CAZyme complexes and an FN3 domain that is postulated to mediate attachment to the bacterial cell-surface (21, 46). Thus, these extracellular enzymes likely have different modes of interacting with the host. In contrast, NanH is predicted to be localized to the cytoplasm of the bacterium (*i.e.* it lacks any obvious export signals), suggesting it does not interact directly with the host. The biological role of NanH is more likely in processing glycans that are imported with their terminal sialic acids intact. This could be the case considering that NanI and NanJ are less efficient (or unable) to process certain acetylated variants. NanH may play the role of “clean up” after sialylated glycans that are recalcitrant to NanI and NanJ are released extracellularly by other glycoside hydrolases and imported into the bacterium. This would rely on one or more hypothetical endo-acting glycanases that could hydrolyze and thereby release sialylated glycans. There is precedent for such endo-acting enzymes in other *O*-glycan metabolizing bacteria but they remain unidentified in *C. perfringens* (47). We suggest that the context of cellular environment is more important to the biological role of the three enzymes than the nuances in their substrate selectivities.

Regardless of cellular environment and locale in the host, sialidase inhibition is a well-sought therapeutic target for the treatment of viral and bacterial pathogens (48). The neuraminidase inhibitor DANA served as a scaffold to develop compounds for influenza viral sialidases, such as oseltamivir, zanamivir, and peramivir (41, 42, 49) and continual development is necessary to counteract the rapid mutation rate of the viral enzymes. These influenza-directed compounds have also been tested against the sialidases of the bacterial pathogen *S. pneumoniae*, with limited effect (50). While no commercial inhibitors were found to be significantly effective against the *C. perfringens* sialidases in this study (K_i_ values < 1.0 μM), the inhibition of bacterial growth by DANA demonstrated that interfering with the ability of this pathogen to remove terminal sialic acids from glycans is a viable strategy. Indeed, further modifications of DANA at the C9 or C5 positions have been shown to increase selectivity for *Vibrio cholera* over human sialidases (51), which lends further support to this approach. The structural determinants of the activity of the three *C. perfringens* sialidases elucidated herein will enable the design of compounds that can maximize interactions within the active site clefts for the development of more potent, specific inhibitors in the treatment of *C. perfringens* causative disease.

## Experimental procedures

### Gene synthesis and recombinant protein expression in *E. coli*

*C. perfringens* CP4 nucleotide sequences were taken from NCBI (52), analyzed by dbCAN2 (53) and InterProScan (54), and modeled using Phyre2 (55). For *nanI* and *nanJ* genes, Phyre2 models were manually curated with PyMOL (Schrödinger, Inc; https://pymol.org/), and GH33 domain amino acid sequence boundaries were chosen based on these analyses; NanI_256-707_, NanJ_192-639_, referred to as NanI_GH33_ and NanJ_GH33_, respectively. The full-length nucleotide sequence for the *nanH* gene was used for all downstream studies; the *nanH* gene encodes for a GH33 domain without any ancillary domains, but for consistency was referred to as NanH_GH33_. All GH33 nucleotide sequences were codon optimized for expression in *E. coli* and gene synthesized including flanking *NdeI* and *XhoI* restriction sites at the 5′ and 3′ ends, respectively, and a stop codon at the 3′ end (BioBasic Inc). Genes were ordered synthesized into the pET28a vector for recombinant protein expression with an N-terminal His_6_-tag for downstream protein purification. A D62N mutation in the NanH_GH33_ construct was introduced *via* the quick-change method to create a catalytically inactivated version of the enzyme for crystallography.

### Protein production for crystallization

The pET28a plasmids containing the gene fragments encoding NanH_GH33_, NanH_GH33_D62N, NanI_GH33_, and NanJ_GH33_ were transformed into *E*. *coli* BL21 (DE3∗) and grown in 2× YT media supplemented with 50 μg mL^-1^ kanamycin at 37 °C. Once the cultures reached an *A*_600_ of 0.6 to 0.8, production of recombinant protein was induced by the addition of 0.5 mM isopropyl β-D-1-thiogalactopyranoside followed by continued incubation at 16 °C. The cells were harvested by centrifugation at 6000 rpm for 15 min at 4 °C. The collected cell pellet was resuspended in sucrose solution (25% sucrose, 20 mM Tris–HCl pH 8.0, and 10 mg mL^-1^ lysozyme), before completing the lysis with a solution containing 1% deoxycholate, 0.1% Triton X-100, 500 mM NaCl, 0.2 μg/ml DNase in 20 mM Tris pH 8.0, and 2 mM MgCl_2_.

The produced recombinant protein was isolated by immobilized metal affinity chromatography (STREAMLINE Chelating beads, GE HealthCare). Fractions that contained the protein of interest were determined by SDS-PAGE and pooled. This was concentrated using a stirred ultrafiltration unit with a 10 kDa membrane (Millipore Sigma) before buffer exchanging by dialysis into 20 mM Tris–HCl pH 8.0 with 100 mM NaCl (the buffer used for NanH also included 1 mM dithiothreitol). The concentrated proteins were further purified by size exclusion chromatography (Sephadex S-200) equilibrated in 20 mM Tris–HCl pH 8.0, with 100 mM NaCl buffer. Fractions containing purified protein were concentrated again in a stirred ultrafiltration cell to 20 mg mL^-1^.

### Crystallization and optimization

NanH_GH33_, NanH_GH33_D62N, and NanJ_GH33_ proteins (20 mg mL^-1^) were crystallized by sitting drop vapor diffusion method at 18 °C. The crystallization conditions for NanH_GH33_ and NanJ_GH33_ were screened by mixing a 1:1 ratio of purified protein with a range of crystallization conditions found in the MCSG 1 to 4 (Anatrace) and Index (Hampton Research) crystallization screens. NanH_GH33_ and the mutant crystallized in the condition 0.1 M Hepes:NaOH pH 7.5, and 20% PEG 8000. NanJ_GH33_ crystals were obtained in 0.1 M Hepes, pH 7.5, 2.0 M ammonium sulfate. NanI_GH33_ (15 g L^-1^) was crystallized following the methods described by Newstead *et al.* (28), using the hanging drop vapor diffusion method, with a 1:1 ratio of purified protein to crystallization solution (20% PEG 3350, 0.2 M KNO_3_ pH 7.0). To obtain the NanI_GH33_ complexes with ligand, the protein was cocrystallized with 15 mM of either Neu5,9Ac or Neu5Gc. The substrate and inhibitor complexes of the NanH_GH33_ and NanJ_GH33_ (both WT and catalytic mutants) were obtained by soaking the crystal in 10 mM of the respective monosaccharide ligands for 30 min. The NanH_GH33_D62N complex with SAc_3_LacNAc was obtained by adding compound in solid form to a drop containing a crystal and soaking for ∼30 min. All crystals were cryoprotected using the crystallization condition supplemented with 20% v/v ethylene glycol prior to cooling directly in an N_2_ stream at 100 K.

### Data collection, structure solution, and refinement

Diffraction data were collected on an instrument comprising a Pilatus 200K 2D detector coupled with a MicroMax-007HF X-ray generator, a VariMax-HF ArcSec Confocal Optical System, and an Oxford Cryostream 800. Data sets were processed using HKL2000 (Table S1). Using the coordinates of apo-NanIGH33 (PDB code 2VK6) and Phaser (56) (https://www.phaser.cimr.cam.ac.uk/index.php/Phaser_Crystallographic_Software), the structures of NanH_GH33_, NanI_GH33_, and NanJ_GH33_ were determined by molecular replacement. Initial refinement of these structures was carried out using REFMAC (57) (https://www2.mrc-lmb.cam.ac.uk/groups/murshudov/content/refmac/refmac.html) before structural corrections and the addition of waters were manually done in COOT (58) (https://www2.mrc-lmb.cam.ac.uk/personal/pemsley/coot/). This model was further improved using Phenix.Refine (59). All the data sets were monitored by flagging 5% of all observation as “free” (60). Validation of the model was performed using MolProbity (61). Final refinement and model statistics are given in Table S1.

### Protein production for enzyme assays

Protein construct vectors were transformed into *E. coli* BL21 Tuner (DE3) cells (Novagen). Cells were grown in LB Miller broth containing 50 μg mL^-1^ kanamycin at 37 °C to an *A*_600_ of 0.6 to 0.8, when protein expression was induced by the addition of isopropyl β-d-1-thiogalactopyranoside to a final concentration of 0.5 mM. The cell culture was incubated at 37 °C for 4 h prior to being harvested by centrifugation at 6500*g* for 20 min at 4 °C. Cell pellets were stored at −20 °C until needed.

The cell pellet from 1 L of bacterial culture was thawed and resuspended in 50 ml of lysis buffer (20 mM Tris pH 8.0, 500 mM NaCl, 0.1 mg mL^-1^ lysozyme). Cells were homogenized by sonication for 2 min of 1 s intervals of sonic pulses at an intensity amplitude of 30 (Fisherbrand Model 705 Sonic Dismembrator and probe; Thermo Fisher Scientific). Cellular debris was removed by centrifugation at 17,500*g* for 45 min at 4 °C. The filtrate was loaded onto a HisTrap FF column (Ni Sepharose 6 Fast Flow; Cytiva) and purified by immobilized metal affinity chromatography; recombinant protein was eluted by an increasing gradient 0 to 500 mM imidazole in 20 mM Tris pH 8.0 and 500 mM NaCl. Protein was concentrated by centrifugation with 30 kDa cutoff Amicon Ultra centrifugal concentrators (Millipore Sigma). His_6_-tagged protein was further purified using a HiLoad 16/60 Superdex 200 prep-grade size exclusion column (GE HealthCare) in 20 mM Tris pH 8, 500 mM NaCl, 2% glycerol (the buffer used for NanH_GH33_ also included 1 mM DTT). Pure protein fractions were pooled and concentrated. Protein purification was monitored throughout by SDS-PAGE. All recombinant proteins were used freshly prepared for downstream enzyme activity assays.

### Activity against O-acetylated sialic acids: BSM assay

Neuraminidase specificity on different sialic acid types was determined using BSM as a model substrate based on a published assay (62). Briefly, 250 μg BSM fortified with 0.5 μg ^13^C_3_-Neu5Ac as an internal standard (ISTD) was dissolved in 50 mM sodium acetate buffer, pH 4.5 and combined with 0.1 mU of neuraminidase based on units predetermined by activity on 4-methylumbelliferone-Neu5Ac. Reactions were incubated at 37 °C for 5.5 h and then stopped by removal of enzyme using 10 kDa molecular weight cutoff spin columns centrifuged at 14,000*g* for 10 min. After this, samples were labeled with 400 μl 24 mM N, N-dimethylbenzylamine in 2M acetic acid at 60 °C for 1 h. Immediately following this, a reverse phase extraction was performed to remove excess N, N-dimethylbenzylamine using 500 mg C18 columns preconditioned with 2 ml 80% acetonitrile/0.1% trifluoroacetic acid, followed by 8 ml of ultrapure water. Samples were then applied to columns, washed with 4 ml ultrapure water, and then eluted in 1.2 ml of 50% acetonitrile. Elutions were dried on the speedvac in the dark, after which they were resuspended in 50 μl of 30% methanol and transferred to vials for ultra high-performance liquid chromatography coupled to mass spectrometry analysis using a Phenomenex Kinetex Biphenyl 100 mm × 2.1 mm column with H_**2**_O and methanol mobile phases and an Agilent Technologies 6530 QTOF mass spectrometer operating in positive ion mode with an Agilent Jet Stream electrospray ionization source as previously published (63, 64). MS peak areas were quantitated relative to the ISTD.

### Microarray printing and screening procedure

The synthesis of the acetylated sialosides was as previously reported (37). All the biotinylated compounds were printed on streptavidin-coated glass slides (SuperStreptavidin Microarray Substrate Slides, Arrayit Inc) using a Scienion sciFLEXARRAYER S3 noncontact microarray equipped with a Scienion PDC80 nozzle (Scienion Inc). Individual compounds were dissolved in PBS buffer (10 mM, pH 7.4) at a concentration of 100 μM and were printed in replicates of six with spot volume ∼ 400 pL, at 20 °C and 50% humidity. Each slide has 21 subarrays in a 3 × 8 layout. The slides were stored at 4 °C after printing and were blocked with TSM binding buffer (20 mM Tris·Cl, pH 7.4, 150 mM NaCl, 2 mM CaCl_2_, and 2 mM MgCl2, 0.05% Tween-20, and 1% bovine serum albumin) for 1 h at 4 °C prior to use. The slides were incubated with different sialidases, including NanH, NanI, and NanJ (20 μg/ml in pH 7.4 PBS buffer) at 37 °C for 1 h. Each slide was then washed with TSM washing buffer, TSM buffer, water, and spun dry. The human-Fc tagged BCoV HE was mixed with goat anti-human IgG antibody (Alexa Fluor 647 conjugated, Jackson ImmunoResearch 109–605–008) in a 1:1 ratio with a final concentration of 3 μg/ml in TSM binding buffer and incubated at 4 °C for 1 h. The glass slide was incubated with the premixed solution at 4 °C for 1 h, washed with TSM washing buffer, TSM buffer, water, and spun dry. The washing steps could be carried out with the cassette attached to protect the unused blocks if necessary. The slides were scanned using a GenePix 4000B microarray scanner (Molecular Devices) at the appropriate excitation wavelength with a resolution of 10 μm. Various gains and PMT values were used in the scanning to ensure that all the signals were within the liner range of the scanner’s detector and there was no saturation of signals. The image was analyzed using GenePix Pro 7 software (version 7.2.29.2, Molecular Devices; https://support.moleculardevices.com/s/article/GenePix-Pro-7-Microarray-Acquisition-Analysis-Software-Download-Page). The data were analyzed with an Excel macro (65) to provide the results. The highest and the lowest value of the total fluorescence intensity of the six replicates spots were removed, and the four values in the middle were used to provide the mean value and standard deviation.

### Enzymatic cleavage of oligosaccharides and HPAEC-PAD analysis

Purified GH33 enzymes were screened for activity on 3′-sialyllactose (Biosynth), 6′-sialyllactose (Biosynth), and disialyllactose (Biosynth). Reactions (250 μl) contained 10 nM enzyme, 1 mg mL^-1^ substrate, and 0 or 500 μM DANA in 20 mM phosphate buffer at the optimal pH for each enzyme (NanH_GH33_ at 7.0, NanI_GH33_ at 6.5, and NanJ_GH33_ at 6.8). Reactions were incubated at 37 °C for 60 min, after which the reactions were treated at 95 °C for 10 min to denature the enzyme and terminate the reaction. Samples were then shortly centrifuged at 8000*g* to pellet denatured protein from the product. HPAEC was performed with a Dionex ICS-3000 chromatography system (Thermo Fisher Scientific) equipped with an autosampler as well as a PAD detector. Taken from the soluble fraction of the enzyme digests, the samples were diluted one-fifth and fortified with 100 μM allose as an ISTD and 10 μl were injected onto an analytical (3 × 150 mm) CarboPac PA20 column (Thermo Fisher Scientific) with a CarboPac PA20 guard column (Thermo Fisher Scientific). Samples were eluted at a 0.4 ml min^-1^ flow rate with a background of sodium hydroxide (NaOH; buffer A) and a gradient of sodium acetate (NaOAc; buffer B) (Table S2). The elution was monitored with a PAD detector (standard quadratic waveform). For quantification, a mixture of monosaccharide and oligosaccharide standards were used in a known concentration series with the following concentrations for a 1× solution, and added 100 μM allose ISTD: 100 μM fucose (Fuc), 100 μM glucose (Glc), 100 μM *N*-acetylgalactosamine (GalNAc), 100 μM lactose (Lac), 100 μM 2′-fucosyllactose (2′-FL), 500 μM Neu5Ac, 100 μM 6′-sialyllactose (6′-SL), 100 μM 3′-sialyllactose (3′-SL). Note that higher amounts of Neu5Ac standard were required to produce an approximately equivalent PAD response to those of other saccharide standards with these instrument conditions (*i.e.* absent a postcolumn addition of NaOH). Quantification of monooligosaccharides and oligosaccharides was performed in Chromeleon software (version 6.80, Thermo Fisher Scientific; https://www.thermofisher.com/order/catalog/product/CHROMELEON6) from triplicate HPAEC-PAD injections.

### Substrate reduction assays using CE-LIF

The relative specificities of each neuraminidase for differing sialic acid linkages were screened using 3′-SL and 6′-SL in CE-based assays (66). 8-Aminopyrene-1,3,6-trisulfonate -labeled 3′- and 6′-SL were produced *via* reductive amination and subsequently excess fluorogenic reagents and unlabeled oligosaccharides were removed using tetrabutylammonium bromide-assisted liquid-liquid extraction (67). Each assay was performed directly in CE-LIF vials which contained both 3′-SL and 6′-SL and similarly prepared 2′-fucosyllactose as an internal standard. Reactions were carried out in freshly prepared 50 mM sodium acetate buffer, pH 4.5, at a temperature of 37 °C. To eliminate the trans-sialidase activity of each neuraminidase 1.2 U of β-galactosidase (Megazyme) were added to each reaction. Total reaction volumes were 50 μl for each enzyme. Samples were hydrodynamically injected every 10 min and separated by CE-LIF exactly as previously described with the exception that fused silica capillaries (DigiKey) were used (67). Note that the first recorded time point (after addition of neuraminidase) was 2 min, reflecting the 2 min capillary rinse before sample injection.

### Enzymatic hydrolysis of the chromogenic substrate X-Neu5Ac, and catalysis and inhibition kinetics

pH optima for the three GH33 domains were assayed using the chromogenic substrate 5-bromo-4-chloro-3-indolyl α-D-N-acetylneuraminic acid (X-Neu5Ac) (Sigma-Aldrich). Hydrolysis was measured spectrophotometrically at 630 nm with 10 nM enzyme and 500 μM X-Neu5Ac in 50 mM citrate-phosphate buffer from pH 3 to 8. Assays were carried out at 25 °C in triplicate in 96-well microtitre plates and measured using a Synergy HT plate reader (BioTek). Data analysis was performed in Prism 9 (GraphPad; https://www.graphpad.com/features). Product equivalents were calculated based on the *A*_630_ of the total cleavage of 100 μM X-Neu5Ac by NanH_GH33_, and thereby calculating a conversion factor.

Enzyme kinetics assays were performed with concentrations of X-Neu5Ac ranging from 7.8 μM to 1 mM and inhibitor concentrations from 0 to 100 μM in 20 mM phosphate buffer at the optimal pH for each enzyme (NanH_GH33_ at 7.0, NanI_GH33_ at 6.5, and NanJ_GH33_ at 6.8). Inhibitors were sourced from Sigma-Aldrich: DANA, siastatin B, peramivir, oseltamivir, and zanamivir. The rate of hydrolysis was quantified using *A*_620_ at 25 °C. Enzyme and inhibitor were incubated for 5 min prior to reaction initiation by the addition of the X-Neu5Ac substrate. Triplicate readings were obtained using a SpectraMax iD3 plate reader (Molecular Devices), and a product conversion factor was calculated as for the pH optima assays but measured at 620 nm. The initial rates were determined by fitting a straight line to the linear portion of the progress curves. The resulting rate data were fitted to Michaelis-Menten curves, and K_i_, K_m_, and V_max_ values for the reactions were obtained from simultaneously fitting the uninhibited and inhibited data to the full mixed enzyme inhibition equation in Prism 9 (GraphPad) (Fig. S3). For those inhibitors that appeared to follow competitive inhibition kinetics, the data were then fit to the competitive inhibition equation for determination of K_i_.

### Growth and inhibition of *C. perfringens* on sialylated substrates

*C. perfringens* CP1 was pregrown in Columbia broth for 16 h from a glycerol stock. The following day, the bacteria were subcultured into 5 ml Columbia and grown for an additional 2 h, after which were pelleted by centrifugation at 5000g for 10 min and resuspended in a previously established *Clostridium perfingens* minimalized medium (17). Cultures were adjusted to inoculate at *A*_600_ of 0.1 in 200 μl minimalized medium supplemented with filter-sterilized 0.5% 3′SL or negative control of filter-sterilized ultrapure water, and in the absence or presence of DANA at 0.01 mM, 0.1 mM, or 1 mM concentrations. Experiments were conducted in six technical triplicates in 96-well plates in an anaerobic chamber at 37 °C for 24 h, with absorbance recorded at 600 nm using a Stratus plate reader (Cerillo), after which culture supernatants were collected, pooled between technical replicates, and filter-sterilized with a 0.2 μm membrane. A 1/10 dilution of the supernatant was then analyzed by HPAEC-PAD using a Dionex ICS-3000 instrument with a CarboPac PA20 column using the same elution method as above (Table S1). The runs also included 50 μg/ml standards of the oligosaccharides used to assess growth or their monosaccharide and disaccharide constituents to annotate metabolites. Bacterial growth kinetics were calculated using GraphPad Prism for exponential growth with log (population).

## Data availability

The accession codes for the crystal structures are available on the PDB as follows: NanH_GH33_ (apo: 8UB5; Neu5Ac complex: 8UL7; Neu59Ac complex: 8ULE; SAc_3_LacNAc complex: 8UM0), NanJ_GH33_ (apo: 8URL; Neu5Ac complex: 8UVV; Neu5,9Ac complex: 9C20) and NanI_GH33_ (Neu5,9Ac: 8U2A; Neu5Gc: 8U5O).

## Supporting information

This article contains supporting information.

## Conflict of interest

A. B. Boraston is an Editorial Board Member/Editor-in-Chief/Associate Editor/Guest Editor for *JBC* and was not involved in the editorial review or the decision to publish this article. The other authors declare they have no conflicts of interest with the contents of this article.
