# Supplementary material for: A “terminal” case of glycan catabolism: Structural and enzymatic characterization of the sialidases of Clostridium perfringens
Source: J Biol Chem. 2024 Sep 7;300(10):107750. doi: 10.1016/j.jbc.2024.107750 (PMC11525138; doi:10.1016/j.jbc.2024.107750)
Supplement: Supporting Information [file mmc1.docx]

Supporting information

**A “terminal” case of glycan catabolism: structural and enzymatic characterization of the sialidases of *Clostridium perfringens***

Brendon Medley^1^*, Kristin E. Low^2^*, Jackline D. W. Irungu^3^, Linus Kipchumba^3^, Parandis Daneshgar^3^, Lin Liu^4^, Jolene M. Garber^2,3^, Leeann Klassen^2^, G. Douglas Inglis^2^, Geert-Jan Boons^4,5^, Wesley F. Zandberg^3^, D. Wade Abbott^2^, and Alisdair Boraston^1^

* These authors contributed equally to this study.

^1^ Department of Biochemistry & Microbiology, University of Victoria, Victoria, BC, V8P 5C2 Canada

^2^ Agriculture and Agri-Food Canada, Lethbridge Research and Development Centre, Lethbridge, AB, T1J 4B1 Canada

^3^ Department of Chemistry, Irving K. Barber Faculty of Science, University of British Columbia, Kelowna, BC, V1V 1M7 Canada

^4^ Complex Carbohydrate Research Center, University of Georgia, Athens, GA 30602, USA

^5^ Chemical Biology and Drug Discovery, Utrecht University, 3584 CG Utrecht, The Netherlands

**Corresponding authors:** Alisdair Boraston ([boraston@uvic.ca](mailto:boraston@uvic.ca)), D. Wade Abbott ([wade.abbott@agr.gc.ca](mailto:wade.abbott@agr.gc.ca)), and Wesley F. Zandberg ([wesley.zandberg@ubc.ca](mailto:wesley.zandberg@ubc.ca))

**Running title:** Characterization of the sialidases of *Clostridium perfringens*

**Supplementary Table 1: X-ray data collection and structure statistics.**

|  | NanH_GH33_  apo | NanH_GH33_  Neu5Ac | NanH_GH33_  Neu5,9Ac | NanH_GH33_  SAc_3_LacNAc | NanI_GH33_  Neu5Ac | NanI_GH33_  Neu5Gc |
| --- | --- | --- | --- | --- | --- | --- |
| ***Data Collection*** |  |  |  |  |  |  |
| Wavelength (Å) | 1.54178 | 1.54178 | 1.54178 | 1.54178 | 1.54178 | 1.54178 |
| Space Group | C2 | C2 | C2 | C2 | P2_1_2_1_2_1_ | P2_1_2_1_2_1_ |
| Cell Dimensions |  |  |  |  |  |  |
| *a, b, c* (Å) | 93.44, 64.49, 65.75 | 93.86, 64.38, 66.81 | 94.04, 64.26, 66.27 | 93.18, 64.69, 65.58 | 69.48, 72.33, 97.35 | 69.27, 72.31, 97.14 |
| α, β, γ (º) | 90.00,102.71, 90.00 | 90.0, 102.45, 90.0 | 90.00 102.7, 90.00 | 90.00,102.75, 90.00 | 90.00, 90.00, 90.00 | 90.00, 90.00, 90.00 |
| Resolution (Å) | 30.00-1.80 | 23.00-1.85 | 22.9 – 1.89 | 24.00-2.46 | 20 – 1.99 | 20 – 1.50 |
| R_meas_ | 0.094 (0.661) | 0.100 (0.730) | 0.142 (0.443) | 0.083 (0.438) | 0.158 (0.452) | 0.059 (0.292) |
| R_pim_ | 0.046 (0.366) | 0.040 (0.446) | 0.057 (0.263) | 0.037 (0.272) | 0.064 (0.261) | 0.025 (0.189) |
| CC1/2 | 0.991 (0.704) | 0.997 (0.762) | 0.981 (0.881) | 0.993 (0.815) | 0.982 (0.791) | 0.998 (0.897) |
| <I/σI> | 14.9 (2.0) | 15.9 (2.0) | 11.6 (2.3) | 16.1 (2.5) | 11 (3.1) | 23.5 (3.1) |
| Completeness (%) | 99.9 (98.6) | 99.8 (99.8) | 100.00 (99.9) | 99.3 (96.4) | 97.8 (88.7) | 98.3 (83.6) |
| Redundancy | 4.0 (2.9) | 4.3 (2.4) | 4.6 (2.6) | 3.9 (2.0) | 5.1 (2.4) | 4.0 (1.8) |
| No. of reflections | 140406 | 155440 | 141496 | 101754 | 170225 | 313778 |
| No. Unique | 35412 (1757) | 36096 (1823) | 30625 (1493) | 25987 (1267) | 33504(1509) | 77611 (3256) |
|  |  |  |  |  |  |  |
| ***Refinement*** |  |  |  |  |  |  |
| Resolution (Å) | 1.80 | 1.85 | 1.89 | 2.46 | 1.99 | 1.50 |
| R_work_/R_free_ | 0.16/0.20 | 0.18/0.22 | 0.19/0.20 | 0.19/0.24 | 0.21/0.25 | 0.15/0.18 |
| No. of atoms |  |  |  |  |  |  |
| Protein | 2849 | 2849 | 2839 | 2815 | 3409 | 3577 |
| Ligand | 42 ACE, 56 EDO | 21 SIA, 56 EDO, 42 SIA | 24 5N6, 56 EDO | 52 SAc_3_LacNAc, 40 EDO | 4 NO_3_, 2 Na, 24 5N6 | 2 Ca, 28 NO_3_ |
| Water | 274 | 274 | 228 | 129 | 161 | 606 |
| *B*-factors |  |  |  |  |  |  |
| Protein | 19.7 | 19.67 | 21.04 | 34.9 | 24.2 | 14.70 |
| Ligand | 28.25 (ACE), 27.39 (EDO) | 28.24 (SIA), 27.39 (EDO) | 26.72 (5N6), 37.03(EDO) | 46.3 (SAc_3_LacNAc), 40 (EDO) | 35.2 NO_3_, 23.4 Na, 27.9 5N6 | 14.04Ca, 30.7 NO­_3_ |
| Water | 25.80 | 25.80 | 26.50 | 34.6 | 23.8 | 30.67 |
| r.m.s.d |  |  |  |  |  |  |
| Bond lengths (Å) | 0.011 | 0.010 | 0.013 | 0.002 | 0.003 | 0.010 |
| Bond angles (°) | 1.078 | 1.071 | 1.338 | 0.481 | 0.677 | 1.152 |
| Ramachandran (%) |  |  |  |  |  |  |
| Preferred | 97.2 | 96.1 | 96.7 | 95.0 | 95.7 | 95.8 |
| Allowed | 2.5 | 3.6 | 3.0 | 5.0 | 4.3 | 4.0 |
| Disallowed | 0.3 | 0.3 | 0.3 | 0 | 0 | 0.2 |

Values for highest resolution shells are shown in parenthesis.

**Supplementary Table 1 (continued): X-ray data collection and structure statistics.**

|  | NanJ_GH33_  Apo | NanJ_GH33_  Neu5Ac | NanJ_GH33_  Neu5,9Ac |
| --- | --- | --- | --- |
| ***Data Collection*** |  |  |  |
| Wavelength (Å) | 1.54178 | 1.54178 | 1.54178 |
| Space Group | P2_1_ | P6_4_22 | P6_4_22 |
| Cell Dimensions |  |  |  |
| *a, b, c* (Å) | 66.25, 48.80, 73.32 | 171.13, 171.13, 92.84 | 171.21, 171.21, 93.40 |
| α, β, γ (º) | 90.00, 93.85, 90.00 | 90.00, 90.00, 120.00 | 90.00, 90.00, 120.00 |
| Resolution (Å) | 23.00 – 2.20 | 20.00 – 2.50 | 22.00- 2.10 |
| R_meas_ | 0.106 (0.389) | 0.079 (0.474) | 0.081 (0.476) |
| R_pim_ | 0.053 (0.210) | 0.032 (0.250) | 0.031 (0.269) |
| CC1/2 | 0.992 (0.909) | 0.997 (0.878) | 0.935 (0.776) |
| <I/σI> | 12.5 (2.6) | 19.0 (2.0) | 16.7 (2.1) |
| Completeness (%) | 99.9 (99.6) | 99.7 (99.3) | 99.6 (99.9) |
| Redundancy | 3.1 (2.0) | 5.5 (3.4) | 5.3 (2.9) |
| No. of reflections | 73593 | 154539 | 252150 |
| No. Unique | 23654 (1095) | 28058 | 47174 |
|  |  |  |  |
| ***Refinement*** |  |  |  |
| Resolution (Å) | 2.20 | 2.50 | 2.70 |
| R_work_/R_free_ | 0.20/0.25 | 0.21/0.25 | 0.19/0.25 |
| No. of atoms |  |  |  |
| Protein | 3384 | 3459 | 3456 |
| Ligand | 2 Ca | 2 Cl, 21 SIA | 4 EDO, 24 5N6 |
| Water | 80 | 138 | 209 |
| *B*-factors |  |  |  |
| Protein | 32.86 | 41.31 | 35.75 |
| Ligand | 45.97 (Ca) | 46.42 (Cl), 46.97(SIA) | 46.75 (EDO), 61.53 (5N6) |
| Water | 30.95 | 37.14 | 31.74 |
| r.m.s.d |  |  |  |
| Bond lengths (Å) | 0.004 | 0.005 | 0.002 |
| Bond angles (°) | 0.658 | 0.796 | 0.468 |
| Ramachandran (%) |  |  |  |
| Preferred | 95.3 | 93.3 | 95.0 |
| Allowed | 4.7 | 6.5 | 5.0 |
| Disallowed | 0.00 | 0.2 | 0.0 |

Values for highest resolution shells are shown in parenthesis.

**Supplementary Table 2: Gradient conditions used for the HPLC separation of glycans.**

| **Time (min)** | **Flow rate (μL/min)** | **Eluent A (100 mM NaOH) (%)** | **Eluent B (100 mM NaOH, 1 M NaOAc) (%)** | **Eluent C (water) (%)** |
| --- | --- | --- | --- | --- |
| 0 | 400 | 30 | 0 | 70 |
| 10 | 400 | 29 | 1 | 70 |
| 30 | 400 | 0 | 30 | 70 |
| 40 | 400 | 0 | 30 | 70 |
| 45 | 400 | 30 | 0 | 70 |
| 55 | 400 | 30 | 0 | 70 |

**Figure S1: Representative electron density maps for ligands modeled into the sialidase structures.** NanH complexes of A) Neu5Ac, B) Neu5,9Ac, C) Neu5,7,9(α2,6)-LacNAc. NanJ complexes of D) Neu5Ac and E) Neu5,9Ac. NanI complexes of F) Neu5,9Ac and G) Neu5Gc. The blue mesh shows the electron density map as maximum likelihood/σ_a_-weighted 2Fo-Fc maps contoured at 1.0 σ.

**Figure S2: pH optima curves for the *C. perfringens* sialidases.** pH optima for the three GH33 domains of NanH_GH33_, NanI_GH33_, and NanJ_GH33_ were assayed using the substrate X-Neu5Ac. Hydrolysis was measured spectrophotometrically at 630 nm in 50 mM citrate-phosphate buffer pH 3 to 8. Assays were recorded in triplicate, and reaction rates were calculated from the linear portion of the progress curve.

**Figure S3: Michaelis-Menten kinetics for the hydrolysis of X-Neu5Ac substrate in the absence and presence of commercial inhibitors.** Enzyme kinetics assays were performed with concentrations of inhibitor from 0 to 100 μM in 20 mM phosphate buffer at the optimal pH for each enzyme. The initial rates of X-Neu5Ac substrate hydrolysis by the GH33 domains were obtained and fit to the Michaelis-Menten equation for competitive inhibition. Data were obtained in triplicate, and error bars represent the standard error of the mean.
